# Wearable and wireless performance evaluation system for sports science with an example in badminton

**DOI:** 10.1038/s41598-022-21187-3

**Published:** 2022-10-07

**Authors:** Ye-Jin Zheng, Wei-Cheng Wang, Yi-Yang Chen, Wen-Hsin Chiu, Rongshun Chen, Cheng-Yao Lo

**Affiliations:** 1 Department of Power Mechanical Engineering, National Tsing Hua University, Hsinchu, Taiwan; 2 Department of Kinesiology, National Tsing Hua University, Hsinchu, Taiwan; 3 Institute of NanoEngineering and MicroSystems, National Tsing Hua University, Hsinchu, Taiwan

**Keywords:** Mechanical engineering, Rehabilitation

## Abstract

Two capacitive sensing units were designed, fabricated, and embedded into two corresponding fingerstalls through microelectronic and additive manufacturing with flexible materials and ergonomic considerations in this study. The sensing units were routed to an adaptor, which in turn was routed to a transmission port (comprising a signal converter and a Bluetooth module), realizing a wearable and wireless force sensing system for sports science applications as the objective. The collected capacitive signals were converted through a preliminarily established database, indicating local force distributions on finger segments. Practical examinations with badminton actions (forehand cross-net shots) were conducted by players to show the effectiveness of the proposed system as an application example. Statistical and quantified results reflected the visual observations on valid shots (67% and 39% for the professional and amateur players, respectively) and well-controlled racket-holding attitude (19.69% and 35.31% force application difference between the first two segments of the index finger of the professional and amateur player, respectively). These proved that the proposed system outperforms existing similar systems in the market and is able to not only classify players with different skill levels but also distinguish attitude stability and controllability, showing scientific evidence in sports science for the first time.

## Introduction

Force sensors have been proposed and realized in commercial products for posture monitoring. For example, many of them were integrated into an array, forming insoles in shoes^1^. By understanding the distributed pressure during walking, diagnoses can be made for shoe-shaped modifications. Another example is the realization of force sensors in modules, demonstrating applications in healthcare, rehabilitation, and medical aid^2^. In these concepts, electrical signals, such as capacitance^3^, resistance^4^, or inductance^5^, were measured under normal forces as a database. Subsequently, during the application, the detected electrical signals were converted to the normal force that was applied vertically to the sensor. Additionally, users usually receive temporal responses as visualizable and quantifiable signals, thus can project the variation of postures in a time-dependent manner^6^. Although examples show interesting wearable electronic applications, they are designed to monitor major actions. Also, because flexibility is a requirement for these sensors, materials usually involve organic elastomers^7^, which are not yet supported by mass production. The realization of miniaturized sensors with elastomers is challenging, thus limiting their application variety.

In contrast to posture, gestures reflect minor actions. Although it shows monitoring complexity, researchers consider that hand gestures result in different operation efficiencies, showing potential applications in sports science^8^. For example, various products that support gesture monitoring have been proposed in the shapes of gloves or wristbands^9,10^. However, most of them have not yet properly considered the need for practical applications. For instance, although the resistive Grip System (Tekscan, Inc.) supports a wide dynamic window (working range), its wired connection limits not only signal transmission but also comfort^8^. When attaching the sensors to the fingers and palms, additional tape is required. This also shows reliability and stability concerns because the sensors can be seriously damaged during attachment and detachment. To demonstrate the effectiveness of wireless signal transmission with proper attachment, the capacitive FingerTPS (Pressure Profile Systems, Inc.) enclosed sensors into fingerstalls^11^; however, its transmission range is short and its dynamic window is narrow. Furthermore, to the best of our knowledge, the effectiveness of these devices has not yet been statistically evaluated for potential applications, as well as the evidence that gesture monitoring by these devices helps to analyze player performance in sports science has never been systematically reported.

Consequently, in this study, we propose a wearable sensing system that includes capacitive sensing units, deformable fingerstalls, and a transmission port that supports signal conversion and wireless transmission. Compared to the existing systems, the proposed system shows a designed dynamic window, proper detection sensitivity, low profile, fast response, and reasonable attachment method. Additionally, to prove the effectiveness of the proposed system and to scientifically demonstrate its supportiveness in sports science, demonstrations and examinations were performed by professional and amateur badminton players.

This study was conducted using theoretical design, numerical evaluation, fabrication, experiment, analysis, and discussion. Comprehensive studies were performed on practical applications, in which statistical results were summarized as proofs of concept.

## Results and discussion

### Operating principle

Considering that minor actions on fingertips may require sensitive responses, in this study, we introduced a capacitive sensing mechanism, which is also more environmentally stable than resistive and inductive ideas. In a capacitor, its capacitance (*C*) is governed by the permittivity (*ε*), overlapping area (*A*), and distance (*d*) between two electrodes if special conditions, such as the fringe effect are neglected, as described in (1). Although many studies have considered other structural modifications of capacitance by, for example, using porous dielectric^12^, hollow structure^13^, or reducing the rigidity of the solid dielectric^14^, capacitance variations are considered controllable only by those three parameters in most cases. Consequently, if the material property or dimension changes owing to the application of forces, the capacitance changes according to (1). Even though the absolute value of *C* can be referred to, the capacitance change (Δ*C*) resulting from the permittivity change (Δ*ε*), overlapping area change (Δ*A*), or distance change (Δ*d*) under applied forces are also referable. Although simultaneous contributions from these three parameters may exist, most researches considered separate contributions from individual parameters based on the applications. In other words, only one parameter was considered variable and the others constants when a specific sensing function was discussed. Additionally, the material (as well as the permittivity) does not change in general practice.

1 $$C = \frac{\varepsilon \times A}{d}$$

As a result, capacitive sensing relies on Δ*d*, which implies normal force (along the ± *z* direction) applications; or on Δ*A*, which implies shear force (in the *xy*-plane) applications^15,16^ when some premises are established: under normal and shear force applications, Δ*A* and Δ*d* do not appear, respectively. In this study, only Δ*A* is considered because the application only involves a normal force application (players hold rackets and apply forces vertically to the sensors). To demonstrate the effectiveness of the proposed system, two sensing units with identical principles, designs, structures, and materials were prepared on a single substrate. However, the two sensing units were routed to the signal converter (to be explained later) independently with a shared common ground to form a dual-channel architecture for analyses in a time-sequential manner.

In addition to the sensing units embedded in the fingerstalls, an adaptor that connects the sensing units and the transmission port (weighted approximately 123 g) was prepared for operation simplicity (to be explained later). The transmission port contains a dual-channel signal converter (sampling frequency of 200 Hz each) and a Bluetooth module for data acquisition and transmission, respectively. Although the transmission port was an additional weight to the players, it was attached on the fore arm, which was distant from the fingertips. Consequently, the additional weight of the transmission port was considered negligible on the results. The responses were delivered to a remote (computer) server wirelessly for further analysis. The sensing units were preliminarily tested under various normal forces, and thus a force–capacitance (*F*–*C*) or force–capacitance change (*F*–Δ*C*) relationship can be established as a database. Because the capacitance shows a temporal response, the converted force from the database also exhibits temporal behavior. To show application simplicity to the users, in this study, we developed a graphical user interface (GUI), in which temporal responses of capacitance and force are shown for reference.

The players wore the system, performed the designed actions, and the capacitive responses were collected before their corresponding forces were converted through the aforementioned relationship. Users thus straightforwardly understand not only the force they apply but also their distributions on the finger segments over time. This significantly improved the accessibility of the meaningful information of force without showing ambiguous information of capacitance (or capacitance change) to the users, as performed in other existing systems.

**Figure 1:**
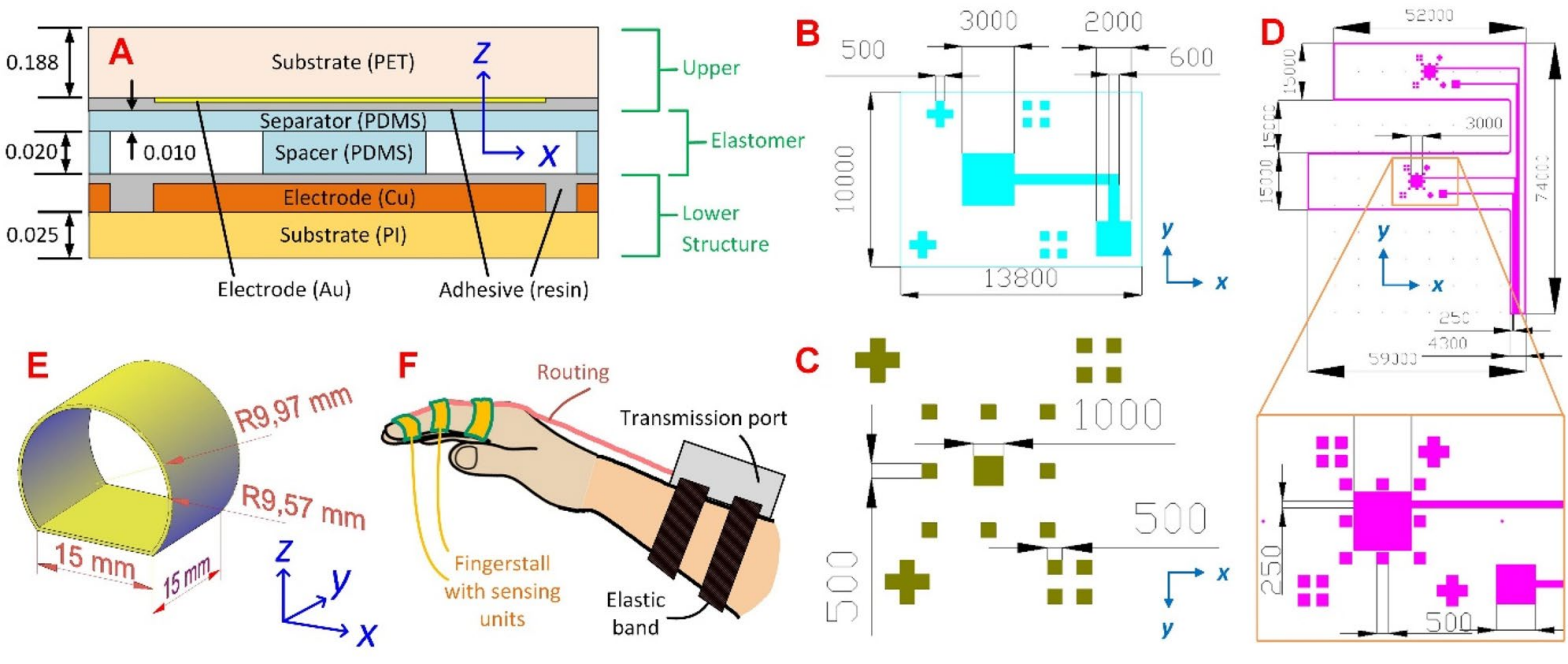
(**A**) The structure of the sensing unit (not to scale). The upper structure contains the upper substrate and the upper electrode, the elastomer structure contains the separator and the spacers, and the lower structure contains the lower electrode and the lower substrate. The top view layout for the (**B**) upper electrode with two types of alignment marks and one connection pad, (**C**) spacers with two types of alignment marks, and (**D**) lower electrode with two types of alignment marks and one connection pad in a single sensing unit. Note that an additional contour was marked in (**D**), which served as the trace for cutting during fabrication. The 3D-printed fingerstall for the (**E**) first segment of the index finger. The fingerstall dimensions were customized for the players hired for the studies conducted in this work. Units in (**A**) and (**E**) are in mm; and (**B**)–(**D**) are in μm.

Although a theoretical capacitor contains only three parameters, as mentioned in (1), there are additional materials and structures involved in practice. For example, the two electrodes cannot float and, thus, should stand on substrates. In another example, the upper electrode should be suspended on the lower electrode and thus supporting spacers should exist. Similarly, charges on the two electrodes neutralize each other, and capacitance does not exist when the two electrodes are in contact. Consequently, an additional separator between the two electrodes is required in all application scenarios. Similar to the findings disclosed in several pieces of literature^17–20^, the capacitor in this study from bottom to top comprised a lower substrate, a lower electrode, adhesive, insulative materials (dielectric air and spacers), a separator, adhesive, an upper electrode, and an upper substrate (Fig. 1A).

### Design and structure of the system

To fulfill the requirement of necessary deformation for force monitoring, soft materials and ductile metals were adopted for these layers. Polyimide (PI) and polyethylene terephthalate (PET) were chosen as the lower and upper substrates, respectively; and Cu and Au were chosen for the lower and upper electrodes, respectively. The selection of Cu and PI facilitates fabrication because existing and standard manufacturing procedures have been established through photolithography. Polydimethylsiloxane (PDMS) was chosen as the spacer and separator because of its noticeable elasticity during deformation^21^. To laminate the different layers, an ultraviolet (UV)-sensitive resin was used.

Because the force applications on the finger pulps of the first two segments of the index finger are being studied, the connections to the lower electrodes of the two sensing units are designed such that routing to the adaptor through the back of the index finger is possible. The upper electrode (Fig. 1B) was isolated by the separator and spacers from its lower counterpart, and the separator and air acted as the dielectric of the capacitor. Additional spacers were arranged at the periphery of the sensing unit (Fig. 1C) to provide uniform support. Owing to the limited spaces located at the bottom of the fingerstalls, the connections to the upper electrodes were made short but were properly routed to corresponding locations on the lower substrate (Fig. 1D). The connection between the lower and upper electrodes was realized using silver paste. Note that additional Cu was arranged on the lower substrate in those spacer locations to ensure identical vertical levels between the spacers and the lower substrate. To laminate these layers, alignment marks were properly arranged on the peripheries of the corresponding structures.

To minimize the impact of the wearable system on player performance, in this study, we aimed to provide ergonomic designs. In comparison to other existing systems that introduce commercialized fingerstalls, we introduced three-dimensional (3D) printing to fabricate them. They were designed in such a way that not only the sensing units can be accommodated but also a comfortable feeling can be assured. To achieve this, thermoplastic elastomer (TPE) was selected as the material and the fingerstalls were designed in tunnel shapes with flat bottoms and different inner bending radii (9.57 mm and 10.25 mm for the first (Fig. 1E) and second segment of the index finger, respectively) for the specific players hired for examinations in this work. The fabricated sensing units were cut into proper dimensions and laminated to the inner side of the fingerstalls (Fig. 1F). During the applications, the flat bottoms of the fingerstalls were arranged in the finger pulps. These fingerstalls improved the practical application challenges on comfort because existing commercialized ones^11,18^ are either too tight to show easy adjustment or too loose to show a reliable response. These fingerstalls also outperformed existing prototype ones^9,10^ because the sensing units can be reused and will not be damaged during attachment and detachment.

It is anticipated that a player can utilize sensing units without affecting the intended actions. Thus, in this study, all the required functions are integrated into a miniaturized transmission port, which can be attached to the player’s arm during application. However, the sensing units and their electrodes were realized on polymeric substrates of PET and PI, which showed substantially different connectors to the coaxial cables that appeared in the signal converter and the Bluetooth module. Hence, to bridge these two interfaces, an adaptor that supports hot swapping (plug-and-play) was adopted. The transmission port comprises three components: lithium (Li)-ion battery, capacitance-to-digital signal converter, and Bluetooth module. The Li-ion battery has a full charge capacity of 90 mAh and lasts for at least one hour, supporting typical uses. The signal converter (Analog Devices, EVAL-AD7152) was responsible for dual-channel data acquisition by a serial communication bus inter-integrated circuit (I2C). The Bluetooth module (SparkFun, ESP32 Thing) was equipped with a microcontroller to transmit the response from the converter to the remote server for further analyses. This architecture not only ensures the expected dual-channel wireless transmission of signals but also potentially supports a modern business model: the sensing units can be easily replaced (detached from the adaptor) as consumables, while the adaptor and the transmission port can be permanently used.

### Evaluation

Although the design, structure, and material used in this study are similar to those disclosed previously, the proposed sensing units must undergo comprehensive evaluation for the dynamic window because it was reported that the normal force applied on the fingertip in a badminton game does not exceed 33 N^22^. As a result, the dynamic window of the proposed sensing unit was designed to support detections up to 30 N, instead of even larger values achieved in other systems. Commercial software (COMSOL Multiphysics, version 5.5) was used for numerical evaluations. The complete structures were modeled, and free triangular shapes were automatically meshed and swept through different layers. This ensured continuous analysis of the materials and structures. A normal force was applied on the top surface of the upper substrate, with the dimensions and projected locations identical to those of the upper electrode. Because the upper electrode as well as the separator and the upper substrate do not further deform when they are in contact with the lower electrode (and the lower substrate), it was expected to exhibit saturation under a large applied force. To obtain the capacitive response, the upper and lower electrodes were set to the terminal and ground, respectively, and a bias of 1 V was applied to the terminal.

**Figure 2:**
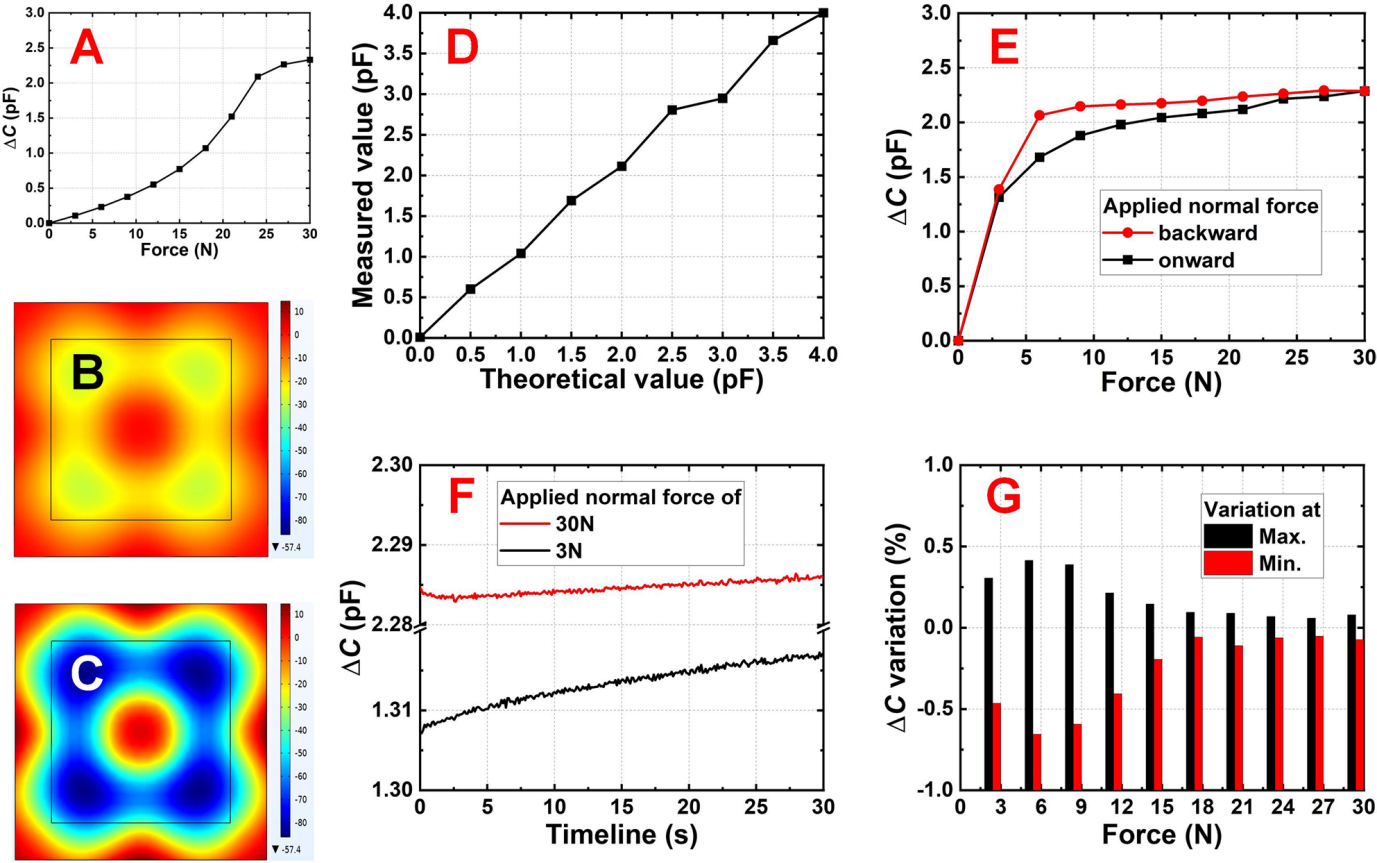
(**A**) The simulated *F*-Δ*C* relationship, in which a positive relationship was observable when the applied force was less than 24 N before signal saturation. The simulated top view structural deformations under (**B**) 10 N and (**C**) 30 N for the sensing unit. Enclosed areas in (**B**) and (**C**) indicate the electrode locations. Color legends in (**B**) and (**C**) are in μm. (**D**) Capacitive response of two commercialized ceramic capacitors in parallel that were sold with 2 pF capacitance values. (**E**) The fabricated sensing unit that shows hysteresis resulted from the elastomeric deformation and recovery. (**F**) The temporal Δ*C* stability when the applied force was low and high, and (**G**) its variation under different applied normal forces which shows the smallest and largest results under 27 N and 6 N, respectively.

As shown in Fig. 2A, the capacitance change along the applied normal force shows not only a positive relationship but also the tendency of expected saturation, indicating a reasonable design. Even though the relationship was nonlinear from a macroscopic viewpoint, which was a result of the serial connection of two capacitors (with a dielectric of PDMS and air)^23^, it shows an expected applicable dynamic window. The major contribution of the capacitance change came from the vertical deformation (in the *z*-direction) of the structure (Fig. 2B and C), which reflects the relationship between Δ*d* and Δ*C*. In addition to the location around the central spacer, which was necessary to support the upper structure, major vertical deformations appeared in the electrode area, as expected. These results showed that the existence of eight peripheral spacers did not hinder structural deformation under applied normal forces. Additionally, regardless of the linearity and absolute values of capacitance change, the results exhibit a one-on-one relationship between the applied normal force and capacitance change, as planned before. Suppose this type of relationship is preliminarily established as a database, the applied normal force can be understood once the capacitance change is known.

The sensing unit was placed above a three-axis stage that supports micro-positioning, which in turn was placed above an optical table for stability. A force gauge that can provide the expected normal force was fixed to the optical table and placed vertically to the sensing unit. A solid acrylic bump was attached to the center of the top surface of the sensing unit to transfer the normal force from the force gauge to the sensing unit. These setups ensured a stable environment for detection and were widely used in similar works^24,25^. The sensing unit is connected to the transmission port through the adaptor, which converts the analog capacitance values to digital ones and delivers the signals to the remote server through Bluetooth. Before the force was applied by the force gauge, a commercially available ceramic capacitor was connected to the transmission port for noise evaluation. The transmission port not only successfully collected the correct capacitance value of the ceramic capacitor, but also less than 1.7% noise was confirmed under 3 N (Fig. 2D). Because the capacitive responses in this study were expected to be less than 3 N, the transmission port supported the required accuracy. To understand the detection sensitivity and response saturation, a stepwise 3 N was provided by the force gauge to the sensing unit when the normal force was lower than 30 N. As shown in Fig. 2E, an *F*–Δ*C* relationship was established, and hysteresis was observed. The hysteresis was caused by the required recovery time of PDMS deformation, which can also be found in related works^26,27^. Nevertheless, because the recovery time of PDMS was announced at approximately 1 s^20^, which was sufficiently short between two consecutive shots in badminton games, in this study, we adopted the onward *F*–Δ*C* relationship as the database and linearly interpolated values in the range of 0–3 N were used. In comparison to the transient response, the long-term stability of the signal is also important. As shown in Fig. 2F, the Δ*C* variation (with respect to the average) was maintained at less than 0.30% for a duration of 30 s under 3 N. Because this duration was sufficiently long for a complete test and the variations were negligible when compared with practical Δ*C* responses (to be explained later), the signal was considered stable. Similarly, the Δ*C* variation was negligible (less than 0.09%) when the applied normal force was strong (30 N). Overall, the peak-to-peak variation was confirmed to be no greater than 0.66% (under 6 N) regardless of the extent of the applied normal force (Fig. 2G).

### Setup and field test

**Figure 3:**
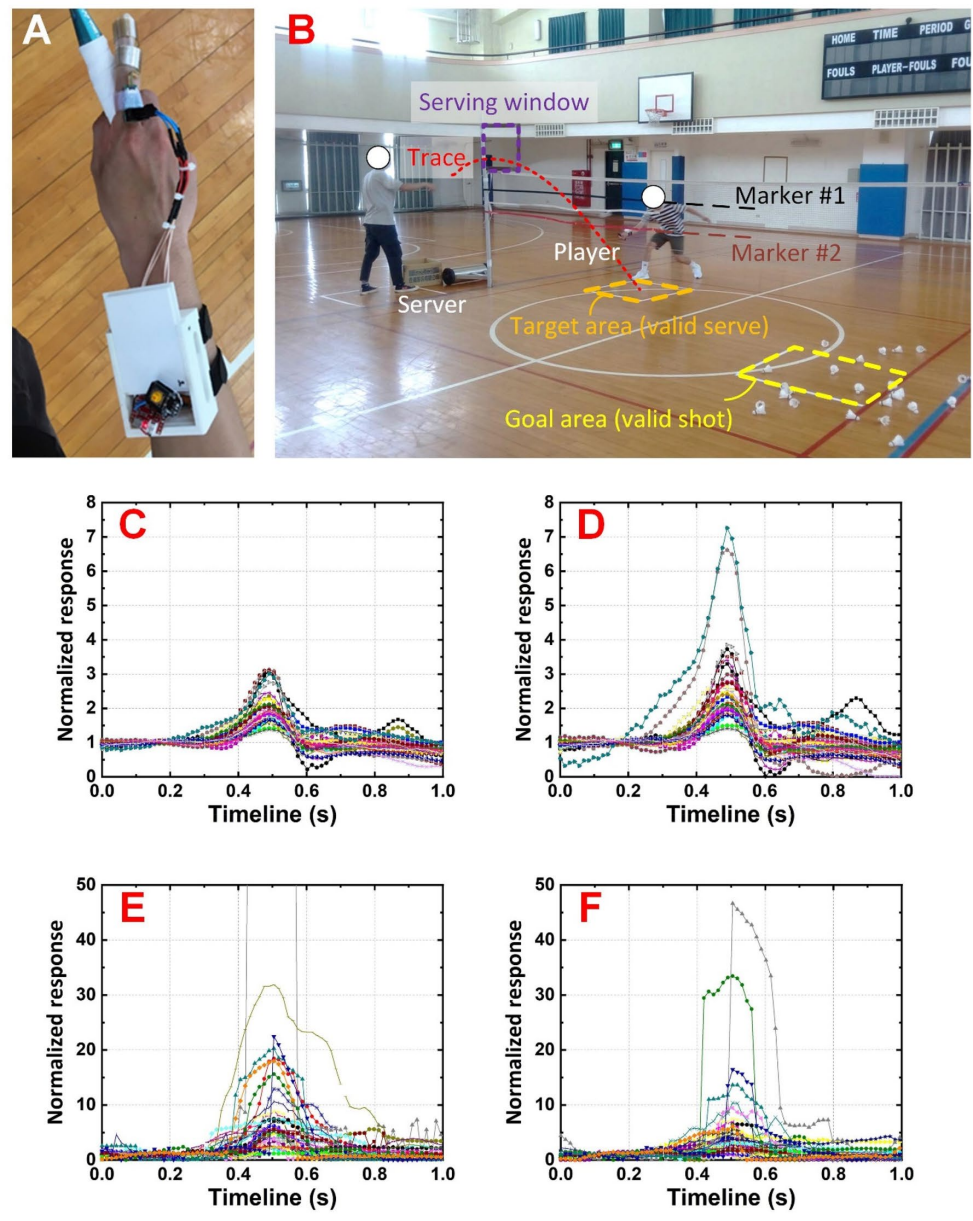
Field test with (**A**) the proposed and fabricated wearable system, in which an additional fingerstall was attached to fix the adaptor and a cover was attached to the transmission port, and (**B**) the court layout, in which target area, goal area, and two markers on the net were indicated for the valid serves and shots. Cumulative 67 normalized capacitive responses on the (**C**) first and (**D**) second segment of the index finger of the professional player. Cumulative 39 normalized capacitive responses on the (**E**) first and (**F**) second segment of the index finger of the amateur player. Note that not only the patterns of the capacitive responses were different but also their absolute values show noticeable difference between the professional and amateur players in (**C**)–(**F**), implying the performance and stability of the players.

A forehand cross-net shot was selected as the action for examination, and two players with different skill levels were hired to conduct the tests. The players wore the developed system (Fig. 3A), and the remote (computer) server was placed outside the court. Additionally, a human being server was hired to serve the shuttlecock through a limited window. In the badminton court, the server and player stood in front of the opposite sides of the net. The court and net were marked to validify serves, and thus, the trace of the served shuttlecock was unified. To synchronize the visual observation and capacitive response in the tests, the high-speed camera recorded the complete action, ensuring valid serves and shots. If the serve was invalid, the test was excluded from the analysis, and 100 valid serves were collected. Only those serves that entered and shots that were conducted in the overlapping space enclosed by the two markers and the cubical target area were counted (Fig. 3B). The players were instructed to return the shuttlecock by the racket to the goal (valid shot) across the net. If the shuttlecock fell outside the goal (even if it was a valid serve), it was considered an invalid shot. The results show that in valid serves, professionals and amateurs showed 67% and 39% valid shots, respectively. Only valid shots were included in the following analyses.

As aforementioned, capacitive responses have been studied in relative (Δ*C*s) instead of absolute values (*C*s). In addition, to highlight the response, normalized Δ*C* (individual Δ*C* divided by the average Δ*C* in a group of Δ*C*s of interest) is widely used in similar studies^13,19^. Considering that the background response (when the player held the racket in static) does not reflect the shot, in this study, we adopted normalized Δ*C* (with respect to the average background Δ*C*s) to unify the expression. Additionally, with the help of a high-speed camera, key capacitive responses were extracted from the complete action of each test. Because the peak response, which indicated the moment of contact between the racket and the shuttlecock, lasted around 0.4 s (appeared in timeline 0.3—0.7 s in Fig. 3C, D, E and F), the response in a duration of 1 s was studied for each test and the peak responses were centered to the timeline of 0.5 s. Consequently, the normalized response was obtained from the original Δ*C*s appeared in the complete 1 s duration divided by the average Δ*C* appeared in timeline 0—0.3 s.

As shown in Fig. 3C, the normalized responses of the first segment of the index finger of the professional player showed noticeable actions. Even though fluctuations appeared before and after the shot, the valid 67 shots performed by the professional player were similar, implying uniform and controllable performance. Similarly, the capacitive responses of the second segment of the index finger of professional player also showed noticeable actions (Fig. 3D). Most of the normalized responses on the second finger segment showed similar values to their first segment counterparts, implying that the racket-holding attitude of the professional player was balanced for the first two segments of the index finger during the actions. In comparison to the results collected through a professional; the responses, which were generated during the valid 39 shots performed by an amateur, exhibited noticeable deviations (Fig. 3E and F). These results imply that the racket-holding attitudes of the professional and amateur players were balanced and unbalanced, respectively.

### Calibration

**Figure 4:**
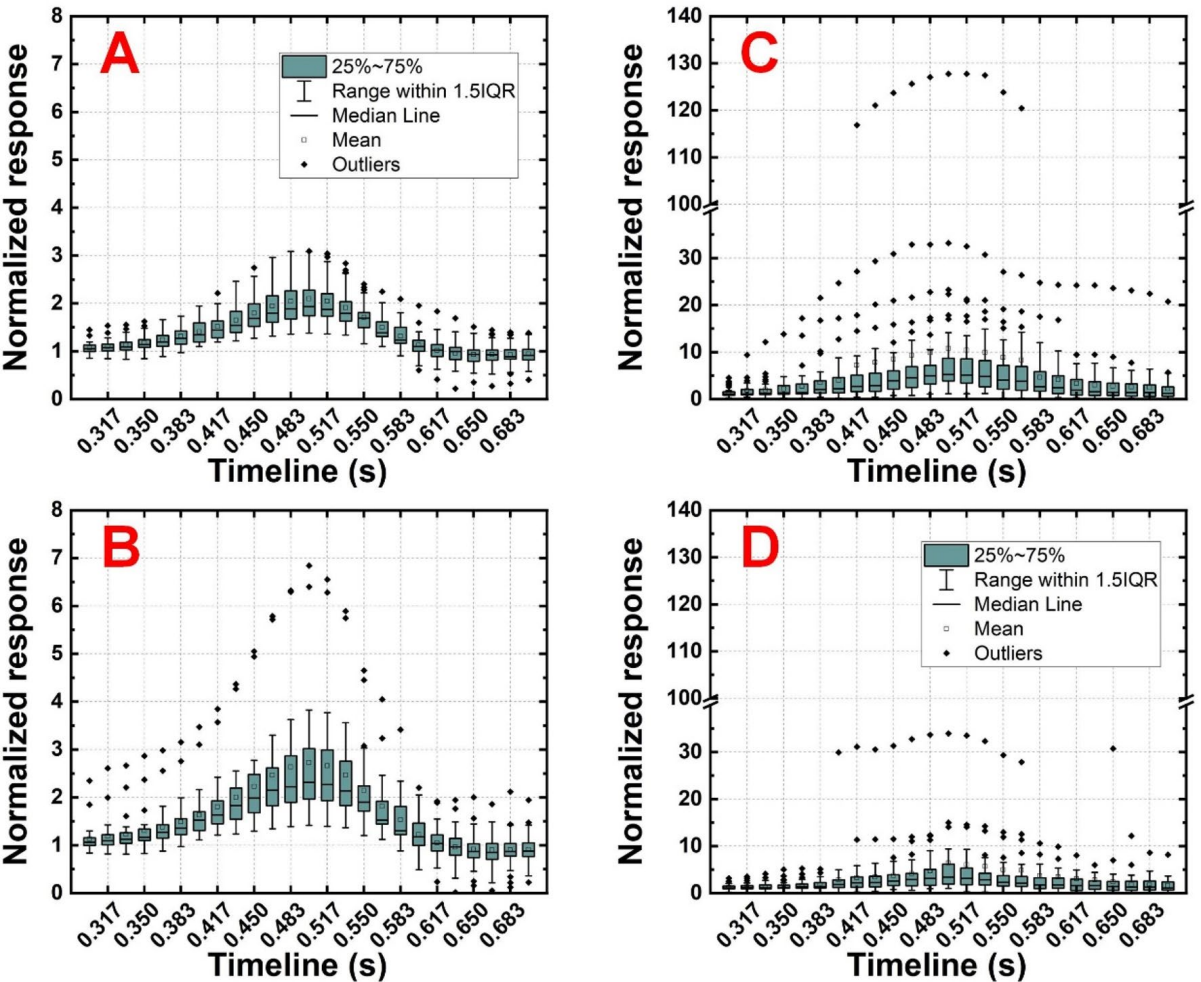
Statistical 67 normalized capacitive responses on the (**A**) first and (**B**) second segments of the index finger of the professional player. Statistical 39 normalized capacitive responses on the (**C**) first and (**D**) second segments of the index finger of the amateur player. All panels share identical statistical definitions and IQR represents interquartile range. Note that the response ranges in (**C**) and (**D**) are different from those in (**A**) and (**B**), and breaks are applied to (**C**) and (**D**).

In addition to the normalized responses from the professional player shown in Fig. 3C and D), another expression of responses can indicate cumulative behavior in mass tests. Here, the normalized response was obtained by dividing the original Δ*C* that appeared in timeline 0.3–0.7 s by the average Δ*C* that appeared in timeline 0–0.3 s. Figure 4A and B is the normalized response expressed statistically from the first and second segment of the index finger of the professional player, respectively. As time elapsed, although the second segment of the index finger showed more outliers than their first segment counterparts, most responses were distributed in limited ranges. Furthermore, the professional player stabilized the racket until the shot and released the holding after the shot, which satisfied the observations of intuitive reactions. Additionally, some values (particularly those after shots) smaller than unity were observed. This was a consequence of the recovery of the elastomer structure used in the sensing unit. When the normal force was released, the upper structure bounced backward (upward) because it was supported by elastomeric spacers and separator. When the distance between the two electrodes increased, the capacitance as well as the normalized Δ*C* decreased, leading to values smaller than unity. Even though the normalized responses from the amateur player shown in Fig. 3E and F did not directly lead to regular patterns compared to their professional player counterparts, statistical distributions of valid shots indicated the instability of the racket-holding attitude of the amateur player (Fig. 4C and D). There were more outliers, and many values were smaller than unity, regardless of either the segment of the index finger or the timeline. This implies that the amateur player could neither stabilize the racket before the shot nor maintain a constant action throughout the mere 0.4 s shot duration.

The peak normalized response (at 0.5 s) on average was 2.09 and 2.72 times higher than the background response in the first and second segment of the index finger of the professional player, respectively, which exhibited a 30.14% difference. Similarly, that was 10.76 and 6.42 times higher than the background response in the first and second segment of the index finger of the amateur player, respectively, which exhibited a 40.33% difference. Even if those outliers were partially excluded and the medians were considered, the professional and amateur players still exhibited 19.69% and 35.31% differences between the two segmental responses, respectively. These results imply that the professional player, compared with the amateur player, not only uniformly held the racket during a single test but also maintained similar attitudes throughout the tests. These results also imply that the amateur player unfortunately not only failed to show stabilized attitudes during tests but also failed to show controllable holding in a short period. Considering that the professional and amateur players had 67 and 39 valid shots, respectively, but comparatively limited and distributed responses, respectively; it was implied that the professional player exhibited superior racket control over the amateur player. These examinations proved the effectiveness and eligibility of the proposed system in distinguishing players with different skill levels. These proofs-of-concept also imply that efficient training and skill monitoring can be expected when personalized databases and programs are customized.

## Method

A 188 μm-thick PET was cleaned properly to serve as the upper substrate. Photolithography, which contained photoresist coating, soft baking, exposure, photoresist development, Au sputtering, and lift-off was conducted with a photo mask that showed patterns of the upper electrodes. After these procedures, the upper structure (upper substrate and upper electrodes inclusive) was ready for assembly.

In contrast to the upper electrode fabrication that deposited metal on the substrate, the lower electrodes were fabricated through an etching process, and a commercially available 18 μm-thick Cu foil plated on a 25 μm-thick PI was prepared. By coating the photoresist onto Cu, exposing the photoresist under UV light through the photo mask, and developing the photoresist, consecutive wet etching in the etchant and a cleaning process in deionized water resulted in the lower electrodes. After these procedures, the lower structure (lower substrate and lower electrodes inclusive) was ready for assembly.

Molding and demolding PDMS are required to generate the designed spacers. A silicon wafer was patterned through photolithography with a photo mask that shows the spacers. The photoresist left on the Si wafer became the hard mask for continuous reactive ion etching, which removed Si and left 20 μm-deep holes. These holes correspond to the spacers after PDMS molding. To facilitate PDMS demolding, anti-adhesion trichloro (1H, 1H, 2H, 2H-perfluorooctyl) silane (Alfa Aesar, L16606) was coated onto the Si after hard mask removal. PDMS was then prepared with a base:hardener = 10:1 ratio, spin-coated onto the Si, and solidified properly. These steps ensured that both the spacers and the separator connected to the spacers were simultaneously fabricated as a whole. After these procedures, the elastomer structure (spacers and separator inclusive) is ready for assembly.

**Figure 5:**
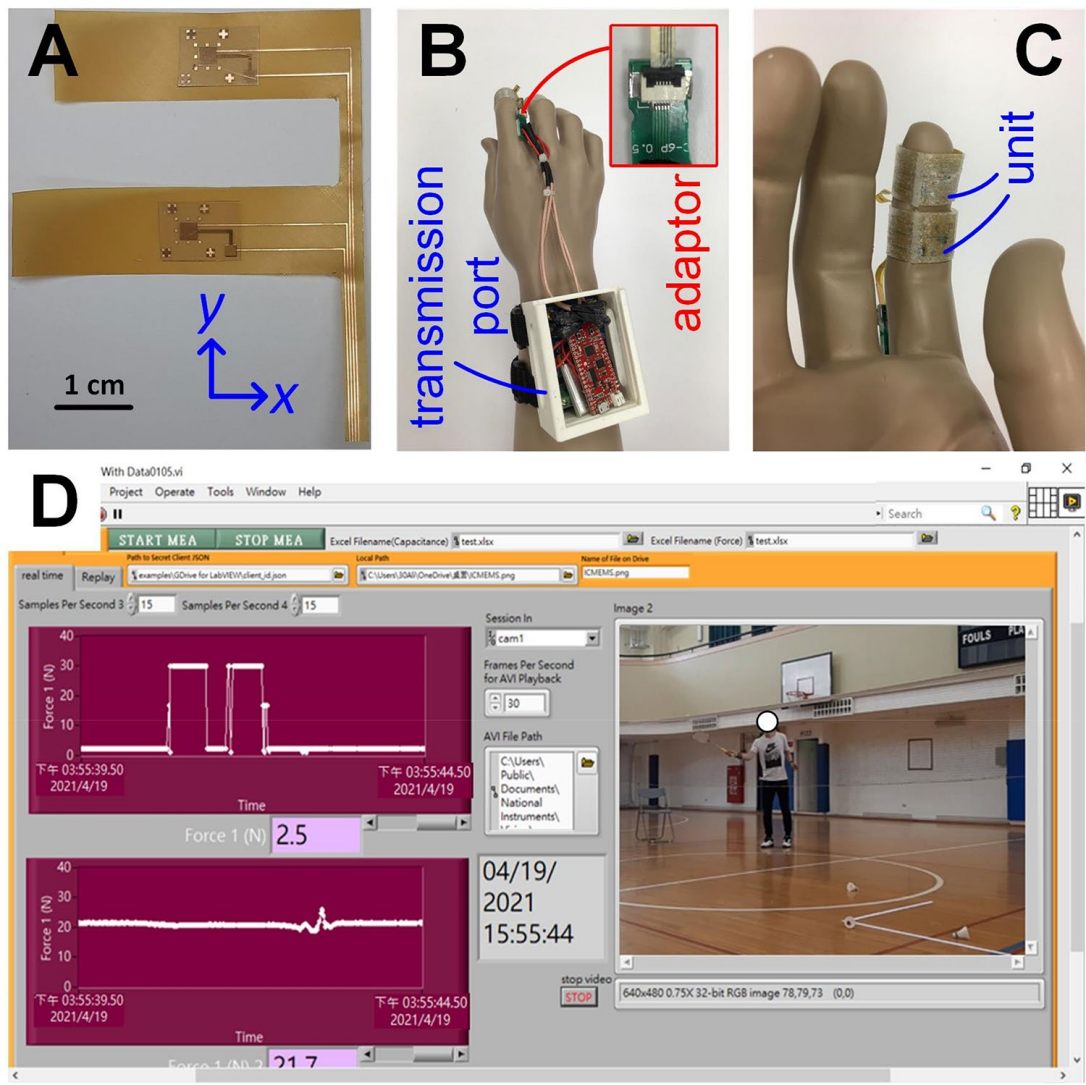
The fabricated (**A**) dual-channel sensing units that contain identical materials, dimensions, and structures; and (**B**) wearable demonstration with the transmission port (3.8 × 6.2 × 7.8 cm^3^) that contains a Li-ion battery, a capacitance-to-digital signal converter, and a Bluetooth module on a plastic model hand through an adaptor (inset) with (**C**) close-up look of the sensors from the finger pulp side. (**D**) The developed user mode GUI for action examination through the high-speed camera (right) and data collection on the first (top left) and second (bottom left) segment of the index finger.

The aforementioned upper structure (the electrode side) was spin-coated with resin, which acted as the adhesive during the first lamination, and aligned to the elastomer structure (the separator side) through alignment marks before the first UV exposure for solidification. The upper and elastomer structures were carefully demolded from Si and ready for the second lamination. The aforementioned lower structure (the electrode side) was also spin-coated with resin and aligned to the demolded elastomer structure (spacer side). A second UV exposure finished the second lamination, and the sensing units were ready to be integrated into the fingerstalls (Fig. 5A).

3D printing by fused deposition modeling was used to fabricate the fingerstalls, in which commercially available TPE was selected as the raw material. The flat bottoms were 15 mm × 15 mm squares, which can accommodate the fabricated sensing units. The rest parts of the fingerstalls were with outer bending radii of 9.97 mm and 10.65 mm, respectively for the first and second segments of the index finger. These dimensions were chosen to fit the sizes of the first two segments of the index finger of the players who underwent the examinations. The size of the fingerstall can be customized for different users and applications, which does not require additional modification of the sensing unit and does not leave potential issues to signal responses.

The sensing units and their connections were properly fixed to two separate fingerstalls; however, the routings were integrated to show a single connection interface to the adaptor (Fig. 5B). Owing to the flexibility of the lower structure of the sensing unit, routings were rolled to the inner tops of the fingerstalls, while the sensing units were placed at the bottom of the fingerstalls (Fig. 5C). The routings went through the backside of the index finger, providing better wearability and reliability compared to existing systems. A third fingerstall, which held the adaptor on the backside of the third segment of the index finger (which is not shown in Fig. 5B), was also prepared. In this work, the weight of the fingerstalls (approximately 1 g) was considered negligible during applications.

To smoothly collect responses and exhibit flexibility in data analyses, a customized GUI was prepared for this study. User and engineering modes were coded based on the commercial software LabVIEW. Additionally, a real-time video captured by a high-speed camera also appeared in the GUI (Fig. 5D). With this, users can examine temporal responses and understand whether the responses came from valid or invalid actions or tests. Raw data can thus be collected through this GUI and saved on a remote server for further analyses.

Throughout this study, no experiments were conducted directly on participants and no identifiable information could be captured by motion data. All participants agree to publish the data included in this work.

## Conclusion

An integrated and wearable force sensing system that contains two capacitive sensing units, two corresponding fingerstalls, one adaptor, and one transmission port (signal converter and Bluetooth module inclusive) was proposed, fabricated, and examined in this study. Results obtained from the practical application (forehand cross-net shot in badminton) indicated that macroscopic skill differences between players can be classified as well as microscopic racket-holding attitude stability difference between two segments of the index finger can be distinguished regardless of the player. These results reflected actual performances, in which valid shots out of valid serves for the professional and amateur players were 67% and 39%, respectively. These statistical and quantified results scientifically prove the effectiveness of the proposed system and the eligibility for similar sports science applications for the first time. The system outperformed other existing systems or proposals, in which results failed to link technical signals to performances or skills. Although this study only demonstrated a two-channel system on the designated index finger with specified players as proofs-of-concept, we considered that this demonstration a stepping stone for related researches. We also expect that the system can be advanced to support multi-channel force detection, detection on all fingers, analyses on other racket sports, and special application such as force monitoring during rehabilitation in the future.

## Data Availability

The datasets used and/or analysed during the current study available from the corresponding author on reasonable request.
